# Supplementary material for: Research on the evaluation method of agricultural intelligent robot design solutions
Source: PLoS One. 2023 Mar 23;18(3):e0281554. doi: 10.1371/journal.pone.0281554 (PMC10035750; doi:10.1371/journal.pone.0281554)
Supplement: S1 File — (DOCX) [file pone.0281554.s001.docx]

**Supporting information (Schedule)**

S1 Table. AHP method to calculate the weights in detail Results

| Guideline layer weights | Sub-criteria layer weights | Weighting value (AHP) |
| --- | --- | --- |
| M  (0.5278) | Drive steady (H_1_) | 0.0249 |
|  | Data Processing (H_2_) | 0.1433 |
|  | Identifying pests (H_3_) | 0.0446 |
|  | Information Collection (H_4_) | 0.2239 |
|  | Remote operation (H_5_) | 0.0911 |
| O  (0.3325) | Image Interaction (H_6_) | 0.0126 |
|  | Intelligent avoidance (H_7_) | 0.0482 |
|  | Terrain adaptation (H_8_) | 0.0786 |
|  | Reasonable structure (H_9_) | 0.1394 |
|  | Button layout (H_10_) | 0.0184 |
|  | Automatic navigation (H_11_) | 0.0353 |
| A  (0.1396) | Fluent modeling (H_12_) | 0.0609 |
|  | Color coordination (H_13_) | 0.0067 |
|  | Anti-theft system (H_14_) | 0.0173 |
|  | Preset work (H_15_) | 0.0442 |
|  | Durable material (H_16_) | 0.0105 |

S2 Table. Detailed calculation results of entropy method and information entropy value

| Evaluation Indicators | Entropy of information($e_{j}$) | Weighting value (Entropy method) |
| --- | --- | --- |
| H_1_ | 0.9282 | 0.0638 |
| H_2_ | 0.8640 | 0.1208 |
| H_3_ | 0.9062 | 0.0833 |
| H_4_ | 0.8830 | 0.1039 |
| H_5_ | 0.7834 | 0.1924 |
| H_6_ | 0.9500 | 0.0444 |
| H_7_ | 0.9478 | 0.0464 |
| H_8_ | 0.9592 | 0.0363 |
| H_9_ | 0.9533 | 0.0415 |
| H_10_ | 0.9460 | 0.0479 |
| H_11_ | 0.9570 | 0.0382 |
| H_12_ | 0.9584 | 0.0370 |
| H_13_ | 0.9592 | 0.0362 |
| H_14_ | 0.9684 | 0.0281 |
| H_15_ | 0.9501 | 0.0443 |
| H_16_ | 0.9600 | 0.0355 |
