# Supplementary material for: Research on the evaluation method of agricultural intelligent robot design solutions
Source: PLoS One. 2023 Mar 23;18(3):e0281554. doi: 10.1371/journal.pone.0281554 (PMC10035750; doi:10.1371/journal.pone.0281554)
Supplement: Attachment — Submitted filename: Response to Reviewers.docx [file pone.0281554.s002.docx]

**Response to Reviewers**

•	Manuscript ID: PONE-D-22-27892

•	Title: “Research on the evaluation method of agricultural intelligent robot design solutions”

Dear Editor:

On behalf of my co-authors, we thank you very much for giving us an opportunity to revise our manuscript, we appreciate editor and reviewers very much for their positive and constructive comments and suggestions on our manuscript entitled “Research on the evaluation method of agricultural intelligent robot design solutions”.

We have studied reviewers’ comments carefully and have made correction which we hope meet with approval. The following are responses to different reviewers' comments.

Reviewer #1:

***Comment:***

The content of the article is consistent with the scientific area of the PLOS ONE. The subject raised by the authors is current and so far rarely noticed by other authors publishing in this area.

The issue described may in the future contribute to improving the efficiency of the automation of the transport process or agricultural intelligent robot ...

The paper has an original, scientific character, related to research on the evaluation method of agricultural intelligent robot design solutions. The authors focus on reducing subjectivity in program selection in the process of designing an agricultural robot and making more objective and rational decisions in order to increase the practicality and scientificity of the program. In addition, the authors correctly propose a new comprehensive evaluation method based on user requirements.

For a better clarification, please edit your paper as follows: 1. Extend the text of the manuscript (e.g. introduction or conclusion) with specific results in the world and Europe, - Improve the quality of the paper by presenting the results of publications by researchers and experts who are involved in this field and are registered in world databases (wos). These are e.g: Simulation of vertical vehicle non-stationary random vibrations considering various speeds, Analysis of stress and strain of fatigue specimens localised in the cross-sectional area of the gauge section testing on bi-axial fatigue machine loaded in the high-cycle fatigue region and Path planning optimization of six-degree-of-freedom robotic manipulators using evolutionary algorithms, thanks.

2. figure 10 should be contrasting and readable,

3. conclusions and future work should be extended to contain practical applications based on research described in this paper - expand references,

4. highlight the course of dependencies/relations in figure No. 1,

5. Unify font in table No: 14.

I recommend publishing the post after the proposed modifications.

***Response:*** Thank you for the useful comments. The changes are highlighted in yellow in 'Revised Manuscript with Track Changes'. It is marked with yellow highlighting.

1. In the introduction section, we have added the publication results of experts in related fields to enhance the quality of the paper.

2. Modified Figure 11 (original Figure 10) by adding annotations of usage scenarios to the figure, as well as comparing it with the traditional assignment method.

3. In the conclusion, the role of the practical applications of the research in this paper in the future is emphasized.

4. Modified Figure 1 to emphasize the process of dependencies in the figure.

5. Standardized the font of Table 14.

Reviewer #2:

***Comment:*** Review report for the paper “Research on the evaluation method of agricultural intelligent robot design solutions”.

Broadly speaking, this paper deals with the evaluation of agricultural intelligent robot design solutions. First, Kano model applied to classify the requirements and establishing an evaluation index system. Secondly, the combination of hierarchical analysis(AHP) and entropy weighting method is used to assign weights to the evaluation index system, calculate the weight value and importance ranking of each index, and carry out various program designs based on the ranking. Finally, the VIKOR method was applied to evaluate and rank the design solutions.

Some typo errors must be rectified. For example, ‘VILOR’ method. Thorough checking is needed.

Significance:-The scientific content of this paper is correct.

-The results could be better presented. This would emphasize the quality of the presented work.

-The limits of the paper are mentioned but some of the points should be investigated.

Quality of presentation:

In eq.(1), the parameter’s range is taken as 0 ≤ ≤ 1. Why is only the decision preference coefficient =0.5 discussed? How do you fix this particular value? What does it signify? Would there be same results if either <0.5 or >0.5. Discuss few cases in each scenario.

Results – When a new evaluation framework is proposed, its result validation and comparisons of the results should be discussed. One can not leave it for future study as the results of the present study are not validated and compared. A sensitivity analysis and comparison are must to include.

Scientific soundness:The subject addressed in this paper is relevant.

Interest to the readers:In my opinion, the method of this paper seems to be interesting for the readership of the journal.

***Response:*** Thank you for the useful comments. Changes are highlighted in pink in 'Revised Manuscript with Track Changes'. It is highlighted in pink.

1. Fixed some grammar and spelling errors.

2. In section 5.3, we add the relevant content about the decision coefficient β, discuss the influence of the value of the decision coefficient on the results under different situations, and determine the value of the decision coefficient of the article based on the publications of related studies and the research needs of this paper.

3. New chapter "5.6 Validation of evaluation results" is added to validate the research results of this paper using fuzzy evaluation method, and the validation results are passed.

Thank you very much for all your help and looking forward to hearing from you soon.

Best wishes.

Ying-Wen Luo
